# Computer assisted diagnosis of Alzheimer’s disease using statistical likelihood-ratio test

**DOI:** 10.1371/journal.pone.0279574

**Published:** 2023-02-17

**Authors:** Xiaoming Zheng, Justin Cawood, Chris Hayre, Shaoyu Wang, for the Alzheimer’s Disease Neuroimaging Initiative Group

**Affiliations:** 1 Medical Radiation Science, School of Dentistry and Medical Sciences, Charles Sturt University, Wagga Wagga, NSW, Australia; 2 Biomedical Sciences, School of Dentistry and Medical Sciences, Charles Sturt University, Wagga Wagga, NSW, Australia; Cardiff University, UNITED KINGDOM

## Abstract

The purpose of this work is to present a computer assisted diagnostic tool for radiologists in their diagnosis of Alzheimer’s disease. A statistical likelihood-ratio procedure from signal detection theory was implemented in the detection of Alzheimer’s disease. The probability density functions of the likelihood ratio were constructed by using medial temporal lobe (MTL) volumes of patients with Alzheimer’s disease (AD) and normal controls (NC). The volumes of MTL as well as other anatomical regions of the brains were calculated by the FreeSurfer software using T1 weighted MRI images. The MRI images of AD and NC were downloaded from the database of Alzheimer’s disease neuroimaging initiative (ADNI). A separate dataset of minimal interval resonance imaging in Alzheimer’s disease (MIRIAD) was used for diagnostic testing. A sensitivity of 89.1% and specificity of 87.0% were achieved for the MIRIAD dataset which are better than the 85% sensitivity and specificity achieved by the best radiologists without input of other patient information.

## Introduction

Alzheimer’s disease is the most common cause of dementia affecting ageing population in the world [1]. MRI T1 weighted structural images are recommended [2] and integrated [3] in routine diagnosis of Alzheimer’s disease (AD). The medial temporal lobe atrophy (MTA) is the hall mark of AD in MRI images [4,5]. Radiologists use a coronal section of the T1 weighted MRI images to rate patients’ MTA using a 5 points visual grading scale, based on the height of the hippocampal formation, the widths of the choroid fissure and the temporal horn [4–6]. A score of 3 or above is considered abnormal [4–6]. A diagnostic sensitivity and specificity of 85% can be achieved by the best radiologists using visual scale grading [6] without using the patients’ other information.

The hippocampus is the mostly affected region among the sub-regions of the medial temporal lobe [4]. Hippocampal atrophy is one of the core biomarkers in the revised National Institute on Aging-Alzheimer’s Association (NIA-AA) diagnostic criteria for AD [7]. In addition to the atrophy of the hippocampal volume, the asymmetry of the left and right hemispheres as well as shapes and forms of the hippocampus are also of great importance [8–10], because differences of these characteristics between AD and normal controls (NC) may point to the origin and staging of the Alzheimer’s disease [11,12]. Such differences may suggest potential targets for therapeutic drugs or directions for any drug development [13,14]. Subfields of the hippocampus have also been investigated in recent years [15].

Many machine learning (ML) algorithms have been developed and studied to assist radiologists’ diagnosis of Alzheimer’s disease using structural MRI images [16,17] including advanced deep machine learning algorithms [18,19]. The diagnostic accuracies of these algorithms are similar to that achieved by radiologists using visual scale rating, i.e. between 80–90% [16,17], or, up to 98.8% if additional information is included [18,19] for distinguishing AD from NC. These computers assisted algorithms are yet to be implemented in clinical practice, particularly, in non-research healthcare settings such as those clinics in regional and remote areas. Clinical radiologists/physicians or healthcare professionals would like to have simple yet accurate tools to assist them in their day-to-day patient care and management. The purpose of this work is to develop a simple computer assisted diagnostic tool in detecting Alzheimer’s disease using an MRI T1 weighted image without the need for knowing any other information of a patient. This is intended to be a convenient and effective tool for assisting radiologists and physicians as well as healthcare professionals such as radiographers or nurses who are caring aging populations in regional and remote areas.

## Methods and materials

Radiologists make diagnostic decisions based on their probability knowledge of normal vs diseased images which were developed during their specialist training [20]. The decision threshold of a human observer can be biased in their decision making and an ideal observer makes decisions by placing a criterion on the axis of an underlying random variable [21]. The statistical likelihood-ratio test of an ideal observer uses the probability density functions of normal vs diseased images in its decision making [21]:

$$L(N)=f_{n}(x)$$ (1)

and

$$L(D)=f_{d}(x)$$ (2)

Where *f_n_*(*x*) and *f_d_*(*x*) are the probability density functions of normal (N) and diseased (D). For a decision variable x, the likelihood ratio observer uses the probability ratio of the normal and diseased or log-likelihood ratio to make decisions:

$$y=logR(D:N)=log\frac{{f(x)}_{d}}{{f(x)}_{n}}=logf_{d}(x)-logf_{n}(x)$$ (3)

Where y>0 is considered to be diseased and y<0 is considered to be normal.

The probability density functions of normal and the diseased can be constructed by using clinical data of pathologically confirmed diseased and normal patients. If the patients are randomly drawn from a population, the probability density functions *f_n_*(*x*) and *f_d_*(*x*) can be considered as normal or Gaussian distributions:

fn(x)=1(2πσn2)12exp[−12[x−μn]σn22] (4)

And

fd(x)=1(2πσd2)12exp[−12[x−μd]σd22] (5)

Where *σ_n_* and *σ_d_* are the standard deviations of normal and diseased and *μ_n_* and *μ_d_* are the mean values of the normal and diseased. The probability density distributions of the normal and diseased can be constructed by using their mean values and standard deviations. On the other hand, if the patients’ data are not normally distributed, the probability density functions of the normal and diseased can be constructed by using freely available statistical package R [22] on the actual data of the patients.

In this work, the probability density distributions of Alzheimer’s patients and normal controls are constructed by using patients’ MRI T1 weighted images which were downloaded from the database of Alzheimer’s disease neuroimaging initiative (ADNI) [23]. All MRI images used in this work are in public domain and the accesses of these databases were approved by the database owners and their respective institutions. As such: (1). the institutional ethics approval was exempted by Charles Sturt University’s Ethics Committee; (2). methods employed in this study are in accordance with the guidelines of Charles Sturt University’s Ethics Committee; and (3). informed consent was obtained prior to data collections by the respective database owners. The ADNI data was collected according to good clinical practice guidelines, US21CFR part 50 –protection of human subjects and part 56 –institutional review boards/ research ethics boards (REBs), and pursuant to state and federal HIPA regulations [23]. The ADNI was launched in 2003 as a public-private partnership, led by Principal Investigator Michael W. Weiner, MD. The primary goal of ADNI has been to test whether serial magnetic resonance imaging (MRI), positron emission tomography (PET), other biological markers, and clinical and neuropsychological assessment can be combined to measure the progression of mild cognitive impairment (MCI) and early Alzheimer’s disease (AD). For multiple images of the same AD or NC patients, we only downloaded one T1 weighted MRI image of each patient for the reason of randomness in constructing our probability density functions. Repeated images of a patient are correlated and their inclusions may increase the numbers of images but reduce the randomness for statistical analysis. A total 526 individual patients’ T1 weighted MRI images (263 AD and 263 NC with randomly mixed gender and ages from 55 to 97 years) were used for the constructions of probability density functions in this study.

**Fig 1:**
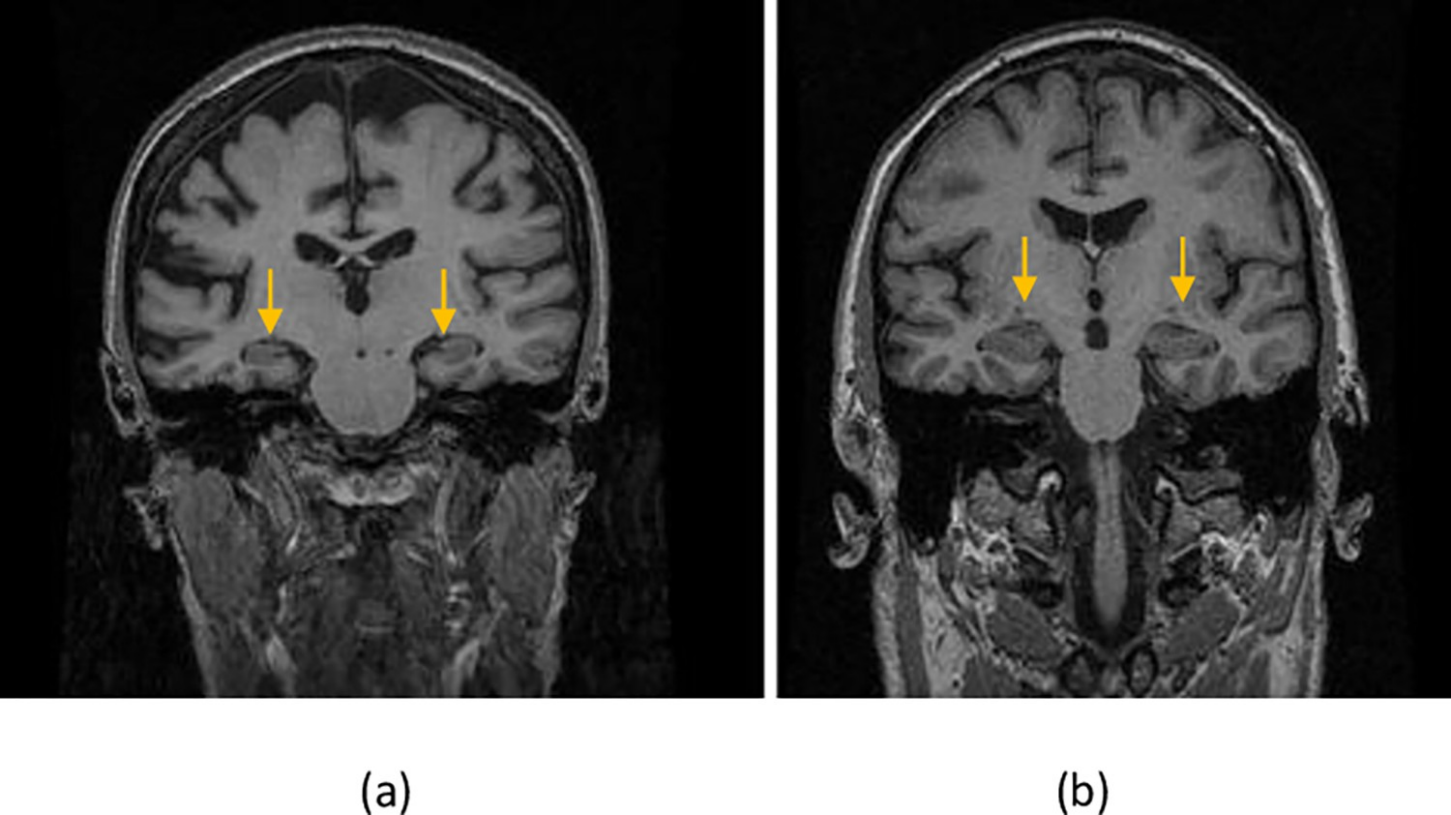
Coronal T1 weighted MRI images of (a) Alzheimer’s patient ID-201 and (b) normal patient ID-200 of the MIRIAD dataset. The FreeSurfer software divides the brain into left and right hemispheres and segments the medial temporal lobe into hippocampus, entorhinal, amygdala and para-hippocampal subregions.

Patients’ hippocampus volumes as well as volumes of other anatomical regions of the brains including sub-regions of the medial temporal lobe were calculated by using the “FreeSurfer” software [24]. FreeSurfer is a software package for the analysis and visualization of structural and functional neuroimaging data from cross-sectional or longitudinal studies. It is developed by the Laboratory for Computational Neuroimaging at the Athinoula A. Martinos Centrer for Biomedical Imaging. It is freely available for public use. As shown in Fig 1, the Freesurfer software divides the brain into left and right hemispheres for various anatomical regions. The medial temporal lobe is segmented into hippocampus, entorhinal, amygdala and para-hippocampal subregions [24]. All T1 weighted MRI images of AD and NC patients were processed by using SPAN computing facility at Charles Sturt University and the National Computational Infrastructure (NCI) of Australia at Canberra. A dataset of Minimal Interval Resonance Imaging in Alzheimer’s disease (MIRIAD) [25] was used for the diagnostic testing. The MIRIAD study was approved by the research ethics committee and written consent was obtained prior to the data collection [26]. The MIRIAD data set consists of a series of T1 weighted MRI images for each of the 46 AD and 23 NC patients. Again, we used one image from each of the 69 individual patients for the reason of randomness. The SPSS statistical package version 25 [27] was used to calculate the area under the curve, Az, or the detectability index, for the joint probability density distributions of normal vs Alzheimer’s disease.

## Results and discussion

Table 1 shows the averaged volumes of left and right hippocampus for both NC and AD patients. The averaged left hippocampal volume is smaller than that of right hippocampus for both AD (97.3 mm^3^) and NC (116.5 mm^3^) subjects. The left/right asymmetry of the hippocampal volumes are 3.26% for AD and 3.20% for NC. There is no statistically significant difference in volumetric asymmetry between AD and NC and no evidence to suggest that the neural degeneration of hippocampus is more or started at the left hand side [11]. Both left and right hand side hippocampal volumes of AD patients are reduced by a similar amount from that of the NC patients (left by 629.5 mm^3^ or 19.4% and right by 648.7 mm^3^, or 19.3%).

**Table 1:** Left and right hippocampus volumes of normal controls (NC) and Alzheimer’s diseases (AD).

	AD		NC	
	Left	Right	Left	Right
Mean Volume (mm^3^) ± SD	2933.4±540.1	3030.7±539.7	3562.9±431.1	3679.4±476.1
Mean Volume (mm^3^) Diff ± SD	-97.3±343.0		-116.5±259.2	
Percentage Differences	3.26%		3.20%	
Correlation (Sig.)	0.798 (0.000)		0.841 (0.000)	

**Fig 2:**
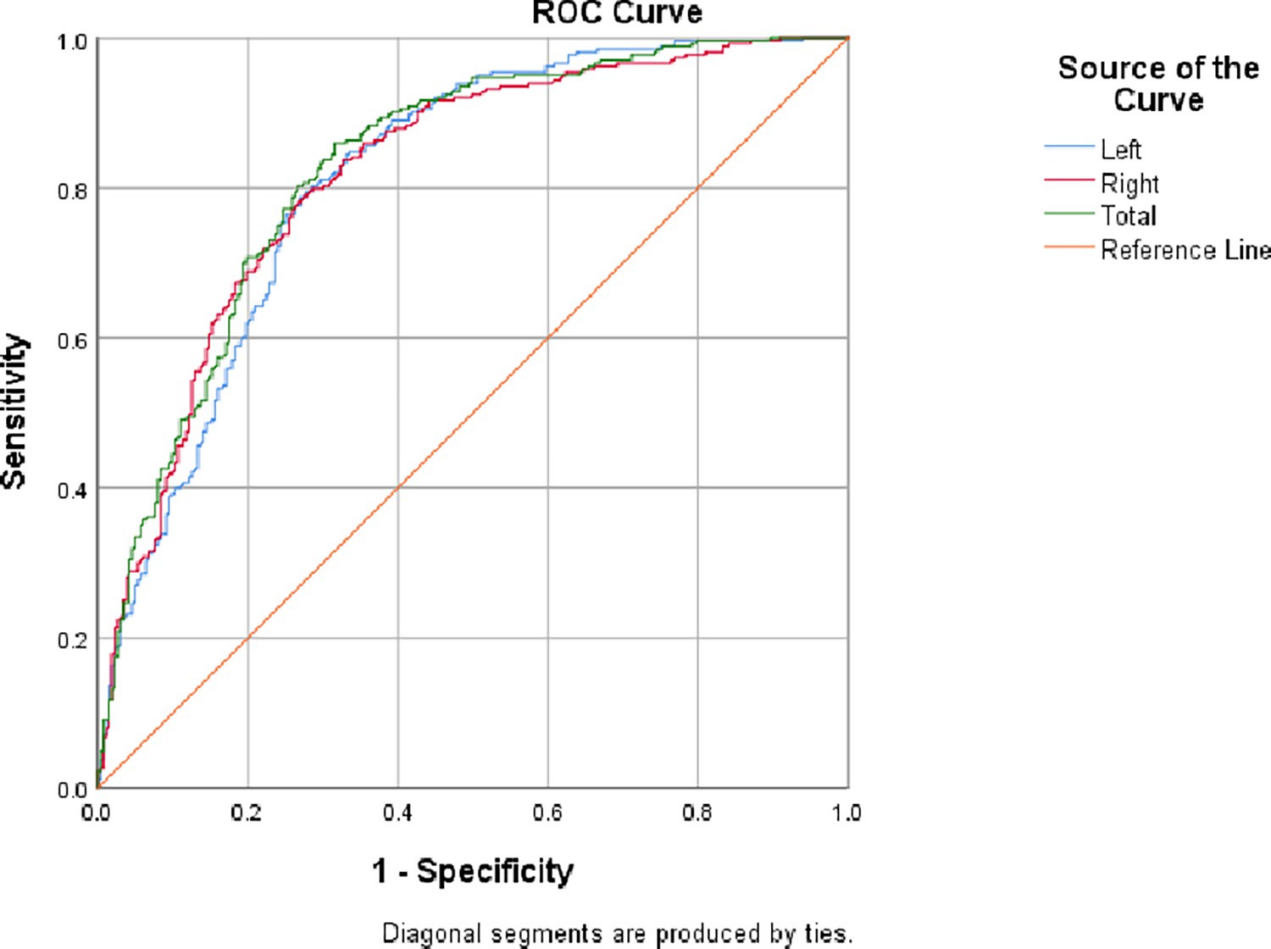
The ROC curves and their areas under the ROC curves of the left, right and total hippocampal volumes for the detection of Alzheimer’s disease.

Fig 2 shows the areas under the ROC curves, Az, or detectability index for the detection of AD by using left and right as well as total hippocampal volumes. The Az equals to 0.814, 0.819 and 0.826 for using left, right and total hippocampal volumes, respectively. The Az values show that using total volumes of hippocampus performed better than that of using either right or left, and using right hippocampal volumes performed better than that of using the left hippocampus. This might be because the averaged right hippocampal volume is slightly larger (3.23%) than that of the left hippocampus.

**Fig 3:**
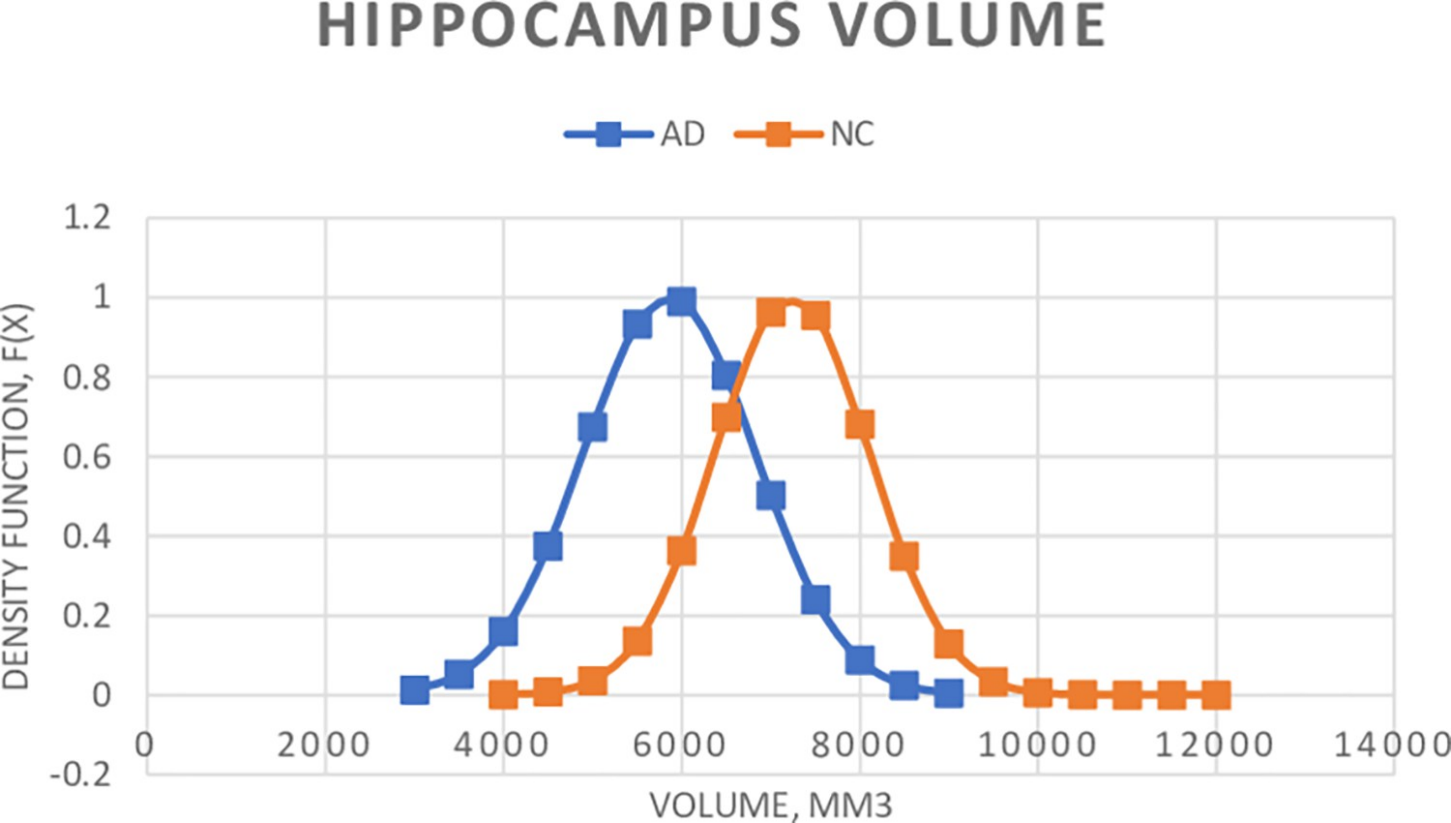
Probability density distributions of total volumes of the hippocampus: Normal controls (NC) vs Alzheimer’s disease (AD). The total mean volumes (±SD) of the hippocampus are 5962.2 (±1025.3) and 7238.4 (±870.0) mm^3^ for AD and NC respectively.

Fig 3 shows the probability density functions of the AD and NC constructed by using the total mean volumes of the hippocampus assuming a Gaussian distribution of Eqs (4 and 5). The total mean volumes of the hippocampus are 5962.1±1025.3 and 7238.4±870.0 mm^3^ for AD and NC respectively. The hippocampal volume of the AD is 19.3% smaller than that of the NC patients on average. To test the randomness of the patient datasets, similar probability density distributions of Fig 3 were also obtained by using the R software on the patients’ data. The results suggest that the Gaussian probability density functions are good for the likelihood ratio observer in detecting Alzheimer’s disease using the hippocampal volumes of the ADNI dataset.

Table 2 shows the mean volumes, standard deviations and detectability indexes of the total medial temporal lobe (MTL) as well as its sub-regions. The volume of the MTL includes hippocampus, amygdala, entorhinal and para-hippocampal volumes. The detectability index or area under the ROC curve for the total MTL volume in detecting Alzheimer’s disease is Az = 0.840 which is the highest among the individual sub-regions of the MTL. The detectability indexes for the individual regions of hippocampus, amygdala, entorhinal and para-hippocampal are Az = 0.827, 0.804, 0.786 and 0.734, respectively, as listed in Table 2. The fact that the detectability index of the total hippocampus is the highest among the indexes of those individual components of MTL is not surprising as the hippocampus atrophy is one of the diagnostic criteria for Alzheimer’s disease [7]. However, the present finding that the total volume of the MTL performed better than individual regions suggests that the AD involves not only the hippocampus but a number of other related sub-regions of the MTL. Our detectability indexes suggest that the para-hippocampal region (Az = 0.734) is the least affected among the four sub-regions of the medial temporal lobe.

**Table 2:** Mean volumes, standard deviations and detectability index Az of various anatomical regions.

	Mean AD	SD AD	Mean NC	SD NC	Az
MTL (mm^3^)	14697.2	2511.1	17943.8	2052.8	0.840
Hippocampus (mm^3^)	5962.1	1025.3	7238.4	870.0	0.826
Amygdala (mm^3^)	2340.9	548.1	2957.1	476.6	0.804
Entorhinal (mm^3^)	3036.7	813.7	3912.4	763.3	0.786
Para-hippocampal	3355.5	616.6	3834.9	518.2	0.734

**Fig 4:**
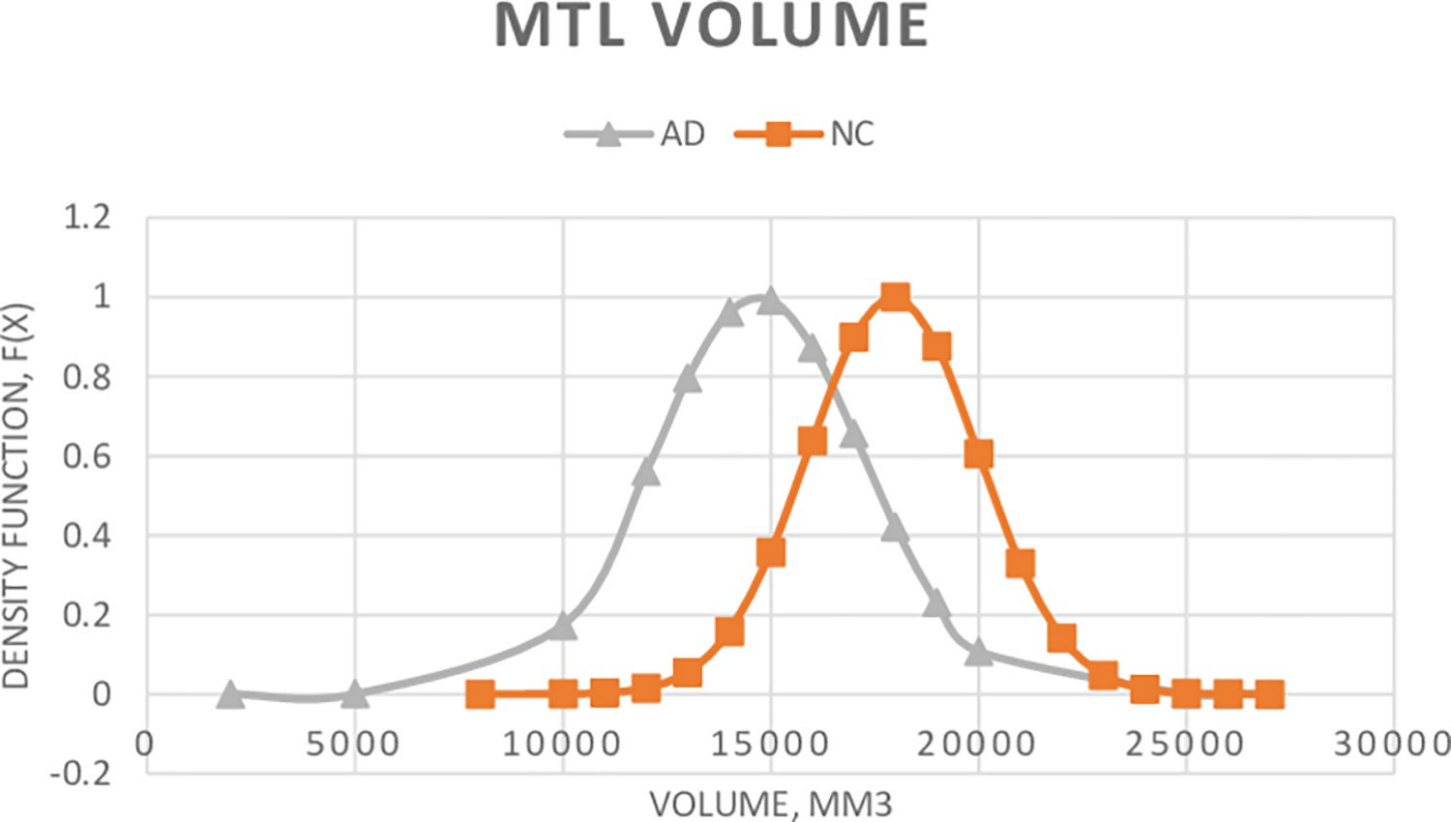
Probability density distributions of total volumes of the medial temporal lobe (MTL): Normal controls (NC) vs Alzheimer’s disease (AD). The mean volumes (±SD) of NC and AD are 17944 (±2053) mm^3^ and 14697 (±2522) mm^3^, respectively.

Fig 4 shows the joint probability density distributions of the medial temporal lobe (MTL) volumes of the normal controls (NC) vs Alzheimer’s diseases (AD). Again, the probability density functions are constructed by using Eqs (4 and 5) as did for hippocampus volumes. The mean volumes of NC and AD are 17944 ± 2053 mm^3^ and 14697 ± 2522 mm^3^, respectively. The likelihood observer uses the joint probability density distributions to calculate the likelihood ratio and determines if the patient should be classified as normal or Alzheimer’s disease. Similar joint probability density distributions can also be constructed for each of the individual anatomical regions, i.e. amygdala, entorhinal and para-hippocampal, and each of these anatomical regions can be used to classify the patents as normal or Alzheimer’s disease. Our detectability analysis for each of the individual sub-regions suggests that the total volume of the MTL is the best for our likelihood ratio observer in the detection of the AD as it has the highest detectability index (Az = 0.840). The MIRIAD dataset [25] was used to test the performances of the likelihood observers constructed by the total volumes of MTL and the hippocampus volumes alone for comparison.

**Fig 5:**
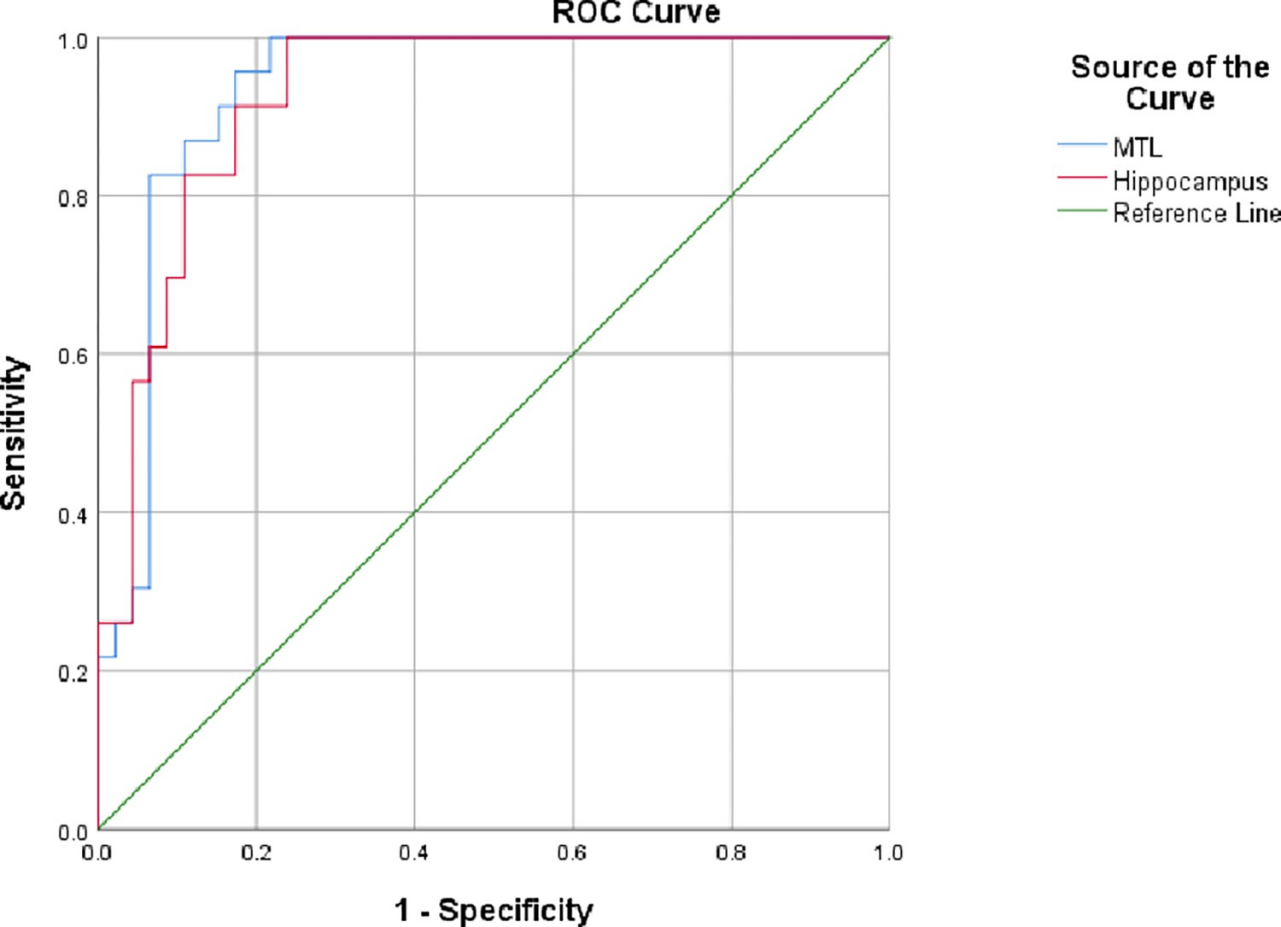
The ROC curves and their areas under the ROC curves for the total MTL and hippocampal volumes for the detection of Alzheimer’s disease.

Fig 5 shows the ROC curves for the MIRIAD dataset by using total MTL and hippocampal volumes. The detectability indexes are calculated to be Az = 0.935 and 0.926 with an upper bound of 0.993 and 0.985 by using the total MTL and hippocampal volumes, respectively. It suggests that the MIRIAD dataset is a good clinical dataset for testing our likelihood ratio observer. Both sensitivity and specificity of the likelihood ratio observer using hippocampus alone are 82.6% whilst the sensitivity and specificity of the likelihood ratio observer using total volume of the MTL are 89.1% and 87.0% respectively. Our result shows that using total volume of the MTL improves the performance by 5.45% in comparison with that of using the hippocampal volumes alone. An 85% of sensitivity and specificity can be achieved by the radiologists using scores of visual grading on the volumes of MTL [6] without other clinical information such as the MMSE scores. Our likelihood observer outperformed the best radiologists and our likelihood observer makes decisions based on MTL volume alone without any input of other patients’ information such as gender or age. Our likelihood ratio observer can, therefore, assist those who do not have specialty knowledge of neuroradiology, which is especially useful for healthcare professionals such as radiographers and nurses caring aging populations in regional and remote areas.

Machine learning (ML) algorithms generally divide a large dataset into subsets for training, validation and testing [28,29]. Our likelihood ratio observer uses the ADNI dataset in constructing the probability density functions which is analogous to the training of ML algorithms or radiologists’ specialist training [20]. The ROC analysis and its detectability index Az are analogous to the validation of ML algorithms using the same ADNI dataset. We used an independent dataset, the MIRIAD dataset, for testing which should be better than that of using the same dataset as that for training (overfitting). It is worth noting that the performance of any machine learning algorithms or Alzheimer’s diagnosis in general is dependent on a number of factors, such as the numbers of patients included in the study and whether or not other biomarkers or variables are included in the diagnostic decision making. By including other biomarkers or variables, the diagnostic accuracy may be improved, i.e. higher than that of using a single biomarker such as the MTL volume. The numbers of patients used in a study can have a major impact on the performance evaluation. The performance index or diagnostic accuracy using smaller numbers of patients may or may not be higher but the performance results from using large numbers of patients should be more robust and reliable [16]. The fact that we used independent (no repeat images of the same patient) 263 AD and 263 NC patients in the construction of the probability density functions for our likelihood ratio observer suggests that the 84% detectability of our likelihood ratio observer using the MTL volume calculated by a publicly available software should be a robust and reliable performance index for any clinical setting without knowing patient’s any other information such as gender or age. Our likelihood ratio observer is equivalent to the best radiologists without any knowledge of neuroradiology. Our likelihood observer needs only a patient’s T1 weighted image to make a diagnostic decision. This is especially useful for healthcare professionals such as radiographers and nurses who don’t have specialist training and are caring aging populations in regional and remote areas. Further work is to test the robustness of our likelihood observer in clinical practice which is underway.

A comparison of our results with a recent work of Ledig et al [17] is worthwhile. Ledig et al [17] used a MAPEM software for brain MR image segmentations and volume calculations. They used both Random Forest and Support Vector Machine (SVM) algorithms for disease classifications. Their highest achievable sensitivities and specificities (Random Forest, SVM) are (83%, 86%) and (90%, 92%), respectively, using all features including gender, brain size and age corrections. This contrasts with our likelihood ratio observer of 87% sensitivity and 89. 1% specificity using MTL volume only without gender, brain size and age corrections. The overall accuracy of our likelihood ratio observer (88%) is equivalent to that of the Random Forest (87%) and SVM (90%) [17]. Using the volumes of hippocampus, entorhinal and amygdala and a linear discriminant analysis (LDA) classifier, the overall classification accuracies of Ledig et al [17] are 78%, 80% and 78% with, and 75%, 73% and 75% without gender, brain size and age corrections. This contrasts with our likelihood ratio observer of 82.6%, 80.4% and 78.6% without gender, brain size and age corrections. Our likelihood ratio observer performed better than that of the LDA using individual anatomical volumes. More importantly, our result is consistent with the fact that hippocampus is the most affected area in Alzheimer’s disease [7] which should have the highest discriminant power for Alzheimer’s disease. It also suggests that the correction for gender, brain size and age could improve the overall diagnostic accuracy by about 3%. One of the main limitations of this work is that gender, brain size and age corrections are not included in the calculations of brain volumes. This limitation is also our strength that the likelihood ratio observer is a simple yet highly accurate diagnostic tool to assist not only radiologists but also allied health professionals such as radiographers and nurses caring ageing populations. Further work is to include age, brain size and gender in the brain volume calculations.

## Conclusions

A computer assisted diagnostic tool is developed and tested for radiologists in their diagnosis of Alzheimer’s disease. It makes diagnostic decision based on a patient’s medial temporal lobe volume which can be calculated by a publicly available software using a T1 weighted MRI images without knowledge of a patient’s any other information such as gender or age. It is a simple and robust tool not only in assisting radiologists for their diagnosis of Alzheimer’s disease, but also for those healthcare professionals such as radiographers or nurses who have no adequate knowledge of neuroradiology and care for aging populations in regional and remote areas. The AD diagnosis by the likelihood ratio observer using hippocampal volume suggests that the hippocampus is the most affected region of atrophy among the sub-regions of the medial temporal lobe but the total volume atrophy of the MTL is more effective in AD diagnosis. There is an asymmetry of the hippocampal volumes that the left is smaller than right on average for both AD and NC patients yet there is no evidence to suggest left/right asymmetrical reductions of the hippocampal volumes between AD and NC patients. Further work is to test the robustness of the likelihood ratio observer in a wider clinical practice for the diagnosis of the Alzheimer’s disease.

## Supporting information

S1 File The dataset for the construction of probability density distribution of Alzheimer’s disease. (ZIP)

S2 File The dataset for the construction of probability density distribution of normal controls. (ZIP)

S3 File The test dataset of Alzheimer’s disease. (ZIP)

S4 File The test dataset of normal controls. (ZIP)
